# Simultaneous pancreas–kidney transplantation in patients with type 1 diabetes reverses elevated MBL levels in association with *MBL2* genotype and VEGF expression

**DOI:** 10.1007/s00125-015-3858-3

**Published:** 2016-01-15

**Authors:** Roel Bijkerk, Pieter van der Pol, Meriem Khairoun, Danielle J. van Gijlswijk-Jansen, Ellen Lievers, Aiko P. J. de Vries, Eelco J. de Koning, Hans W. de Fijter, Dave L. Roelen, Rolf H. A. M. Vossen, Anton Jan van Zonneveld, Cees van Kooten, Marlies E. J. Reinders

**Affiliations:** Department of Nephrology, Leiden University Medical Center, Albinusdreef 2, 2333 ZA Leiden, the Netherlands; Department of Immunohaematology and Blood Transfusion, Leiden University Medical Center, Leiden, the Netherlands; Department of Clinical Genetics, Leiden University Medical Center, Leiden, the Netherlands; Einthoven Laboratory for Experimental Vascular Medicine, Leiden University Medical Center, Leiden, the Netherlands

**Keywords:** Diabetic nephropathy, Mannan-binding lectin, MBL, Simultaneous pancreas–kidney transplantation, Type 1 diabetes, Vascular injury, VEGF

## Abstract

**Aims/hypothesis:**

High levels of circulating mannan-binding lectin (MBL) are associated with the development of diabetic nephropathy and hyperglycaemia-induced vasculopathy. Here, we aimed to assess the effect of glycaemic control on circulating levels of MBL and the relationship of these levels with vascular damage.

**Methods:**

We assessed MBL levels and corresponding *MBL2* genotype, together with vascular endothelial growth factor (VEGF) levels as a marker of vascular damage, in type 1 diabetes patients with diabetic nephropathy before and after simultaneous pancreas–kidney (SPK) transplantation. We included diabetic nephropathy patients (*n* = 21), SPK patients (*n* = 37), healthy controls (*n* = 19), type 1 diabetes patients (*n* = 15) and diabetic nephropathy patients receiving only a kidney transplant (*n* = 15). Fourteen diabetic nephropathy patients were followed up for 12 months after SPK.

**Results:**

We found elevated circulating MBL levels in diabetic nephropathy patients, and a trend towards elevated circulating MBL levels in type 1 diabetes patients, compared with healthy control individuals. MBL levels in SPK patients completely normalised and our data indicate that this predominantly occurs in patients with a polymorphism in the *MBL2* gene. By contrast, MBL levels in kidney transplant only patients remained elevated, suggesting that glycaemic control but not reversal of renal failure is associated with decreased MBL levels. In line, levels of glucose and HbA_1c_, but not creatinine levels and estimated GFR, were correlated with MBL levels. VEGF levels were associated with levels of MBL and HbA_1c_ in an MBL-polymorphism-dependent manner.

**Conclusions/interpretation:**

Taken together, circulating MBL levels are associated with diabetic nephropathy and are dependent on glycaemic control, possibly in an *MBL2*-genotype-dependent manner.

## Introduction

High levels of circulating mannan-binding lectin (MBL) have been demonstrated to be associated with the clinical manifestation of type 1 diabetes [1] and the development of diabetic nephropathy [2]. MBL is an essential component of the innate immune response; the protein is the major recognition molecule of the lectin pathway of complement activation and is activated by binding sugar moieties [3]. Enhanced glycation results in increased MBL activity and the subsequent activation of the complement system [4] and high levels of cytokines specific to type 1 diabetes [5].

Specific single nucleotide polymorphisms (SNPs) in the *MBL2* gene and promoter region result in inter-individual variations in circulating levels of functional MBL (0–4,000 μg/l). The effect of different *MBL2* genotypes on the risk of developing diabetic nephropathy is unclear as some studies have reported that individuals with high-producing *MBL2* genotypes have an increased risk of developing nephropathy [6], whereas others have found that genotype variations do not confer risk of diabetic nephropathy [7].

In a model of type 1 diabetes in mice, MBL deficiency attenuates renal changes [8]. Furthermore, diabetes mellitus is strongly associated with microvascular complications (including retinopathy, neuropathy and nephropathy), and vasculopathy resulting from hyperglycaemia has been shown to be dependent on MBL and lectin complement pathway activation [9].

Simultaneous pancreas–kidney (SPK) transplantation is an advanced treatment option for type 1 diabetic patients with diabetic nephropathy and we previously demonstrated that microvascular damage is reversed in the first year after SPK [10]. Here, we studied this cohort of healthy control participants and type 1 diabetes patients before and following SPK or kidney transplantation alone to investigate the relationship between circulating MBL and vascular endothelial growth factor (VEGF) levels, and how glycaemic control and *MBL2* genotype influence their levels.

## Methods

### Patients and design

All procedures were approved by the institution’s Medical Ethical Committee and written informed consent was obtained from all patients and control participants.

Details of the cohort have previously been described [10, 11] and are presented in Table 1. In short, 107 patients were enrolled in an observational, cross-sectional study that consisted of five groups: (1) a control group (*n* = 19) of healthy volunteers; (2) type 1 diabetic patients with an estimated GFR (eGFR) of ≥30 ml min^−1^ 1.73 m^−2^ (*n* = 15); (3) type 1 diabetic patients with diabetic nephropathy (*n* = 21); (4) diabetic nephropathy patients with a functioning kidney graft after kidney transplantation (KTx, *n* = 15); and (5) patients who received SPK in the past (*n* = 37). Inclusion and exclusion criteria were previously described [10].

**Table 1:** Cross-sectional study patient characteristics

	Controls (*n* = 19)	DM ≥30 ml min^−1^ 1.73 m^−2^ (*n* = 15)	DN (*n* = 21)	SPK (*n* = 37)	KTx (*n* = 15)
Sex, male, *n* (%)	9 (47%)	6 (40%)	16 (76%)	24 (65%)	6 (40%)
Age (years)	44 ± 11	55 ± 13*	44 ± 5^†^	48 ± 8	48 ± 10
BMI (kg/m^2^)	25.2 ± 3.8	23.8 ± 2.8	25.4 ± 3.2	24.3 ± 4.4	25.0 ± 4.6
HbA_1c_ (%) (mmol/mol)	–	7.1 ± 0.7 (54 ± 7.7)	8.9 ± 2.3^†^ (74 ± 25.1)	5.6 ± 0.8^†,‡^ (38 ± 8.7)	8.5 ± 0.9^†,§^ (69 ± 9.8)
Glucose (mmol/l)	5.3 ± 1.0	12.8 ± 4.7*	13.8 ± 6.4*	6.0 ± 2.9^†,‡^	13.0 ± 6.7*^,§^
eGFR (ml min^−1^ 1.73 m^−2^)	93 ± 17	70 ± 24*	18 ± 7*^,†^	53 ± 19*^,†,‡^	62 ± 23*^,‡^
Median proteinuria (g/24 h) (IQR)	–	0.29 (0.13–0.29)	0.72 (0.35–1.5)	0.27 (0.17–0.82)^‡^	0.21 (0.18–0.36)^‡^
Systolic blood pressure (mmHg)	131 ± 12	130 ± 13	146 ± 19	139 ± 23	138 ± 29
Diastolic blood pressure (mmHg)	82 ± 7	71 ± 8*	86 ± 11^†^	83 ± 13^†^	81 ± 14
Haemoglobin (mmol/l)	8.7 ± 0.7	8.2 ± 1.3	7.6 ± 0.5*	8.1 ± 1.2	8.2 ± 1.1
Haematocrit (l/l)	0.41 ± 0.03	0.40 ± 0.05	0.36 ± 0.03*	0.40 ± 0.05^‡^	0.41 ± 0.05^‡^
Duration of diabetes (years)	–	35 ± 10	29 ± 9	27 ± 8^†^	35 ± 9^§^
Dialysis, *n* (%)	–	0 (0%)	3 (14%)	0 (0%)^‡^	0 (0%)
Median time since KTx or SPK (months) (IQR)	–	–	–	45 (19–110)	21 (9–69)
Rejection after KTx or SPK, *n* (%)	–	–	–	13 (35%)	0 (0%)
Diabetes after SPK, *n* (%)	–	–	–	3 (8%)	–
Smoking, *n* (%)	0 (0%)	2 (13%)	0 (0%)	3 (8%)	1 (7%)
Acetylsalicylic acid, *n* (%)	–	3 (20%)	2 (10%)	11 (30%)	3 (20%)
Antihypertensive drugs, *n* (%)	–				
ACE inhibitor		7 (47%)	14 (67%)	14 (38%)	7 (47%)
Angiotensin-II antagonist		3 (20%)	13 (62%)^†^	8 (22%)^‡^	0 (0%)^‡^
Calcium antagonist		2 (13%)	11 (52%)^†^	22 (60%)^†^	7 (47%)
Diuretic		5 (33%)	13 (62%)	9 (24%)^‡^	4 (27%)^‡^
β-Blocker		0 (0%)	9 (43%)^†^	19 (51%)^†^	6 (40%)^†^
Statin, *n* (%)	–	8 (53%)	13 (62%)	26 (70%)	5 (33%)
Steroid-free, alemtuzumab induction, *n* (%)	–	–	–	12 (32%)	1 (7%)
Immunosuppressive drugs, *n* (%)	–	–	–		
Cyclosporine				13 (35%)	1 (7%)^§^
Tacrolimus				24 (65%)	11 (73%)
Prednisone				26 (70%)	9 (60%)
Azathioprine				3 (8%)	0 (0%)
Sirolimus				0 (0%)	1 (7%)
Everolimus				2 (5%)	0 (0%)
Mycophenolate mofetil				27 (73%)	14 (93%)

Parametric data are presented as mean ± SD. Nonparametric data are presented as median and IQR. Categorical data are presented as frequency and percentage

*p < 0.05 vs controls; ^†^ *p* < 0.05 vs DM ≥30 ml min^−1^ 1.73 m^−2^; ^‡^ *p* < 0.05 vs DN; ^§^ *p* < 0.05 vs SPK

ACE, angiotensin-converting enzyme

In a longitudinal study (Table 2), diabetic nephropathy patients who received SPK were followed up (*n* = 14): plasma samples were obtained before (D0) and 1 month (M1), 6 months (M6) and 12 months (M12) after SPK.

**Table 2:** Follow-up study patient characteristics

	D0 (*n* = 14)	M1 (*n* = 14)	M6 (*n* = 14)	M12 (*n* = 14)
Sex, male, *n* (%)	13 (93%)	–	–	–
Age (years)	45.1 ± 5.2	–	–	–
BMI (kg/m^2^)	26.0 ± 2.8	24.7 ± 2.9	24.7 ± 2.1	24.8 ± 2.5
HbA_1c_ (%) (mmol/mol)	8.8 ± 1.7 (73 ± 18.6)	6.5 ± 1.8* (48 ± 19.7)	5.3 ± 0.3* (34 ± 3.3)	5.4 ± 0.2* (36 ± 2.2)
Glucose (mmol/l)	14.7 ± 7.1	6.3 ± 1.0*	5.3 ± 1.4*	5.8 ± 1.5*
eGFR (ml min^−1^ 1.73 m^−2^)	18 ± 9	54 ± 19*	54 ± 15*	54 ± 11*
Median proteinuria (g/24 h) (IQR)	0.73 (0.36–1.30)	0.66 (0.28–1.15)	0.41 (0.17–0.98)	0.37 (0.14–1.10)
Systolic blood pressure (mmHg)	153 ± 15	129 ± 21*	133 ± 20*	131 ± 14*
Diastolic blood pressure (mmHg)	87 ± 11	78 ± 11	78 ± 11	78 ± 6
Haemoglobin (mmol/l)	7.6 ± 0.5	6.6 ± 0.9*	7.4 ± 0.9	8.0 ± 1.0
Haematocrit (l/l)	0.37 ± 0.03	0.33 ± 0.05	0.37 ± 0.04	0.40 ± 0.05
Diabetes after SPK, *n* (%)	–	1 (7%)	2 (14%)	0 (0%)
Smoking, *n* (%)	0 (0%)	0 (0%)	0 (0%)	0 (0%)
Acetylsalicylic acid, *n* (%)	3 (21%)	1 (7%)	1 (7%)	4 (29%)
Antihypertensive drugs, *n* (%)				
ACE inhibitor	9 (64%)	2 (14%)*	3 (21%)*	3 (21%)*
Angiotensin-II antagonist	9 (64%)	1 (0%)	1 (0%)	1 (0%)
Calcium antagonist	8 (57%)	4 (29%)	5 (36%)	8 (57%)
Diuretic	10 (71%)	1 (0%)	1 (7%)*	2 (14%)*
β-Blocker	8 (57%)	6 (43%)	4 (29%)	4 (29%)
Statin, *n* (%)	8 (57%)	2 (14%)*	2 (14%)*	3 (21%)*
Steroid-free, alemtuzumab induction, *n* (%)	–	14 (100%)	–	–
Immunosuppressive drugs, *n* (%)	–			
Cyclosporine		1 (7%)	2 (14%)	1 (7%)
Tacrolimus		12 (86%)	11 (79%)	11 (79%)
Prednisone		1 (7%)	4 (29%)^†^	4 (29%)^†^
Everolimus		1 (7%)	1 (7%)	2 (14%)
Mycophenolate mofetil		14 (100%)	13 (93%)	14 (100%)

Parametric data are presented as mean ± SD. Nonparametric data are presented as median and IQR. Categorical data are presented as frequency and percentage

*p < 0.05 vs D0; ^†^ *p* < 0.05 vs M1

ACE, angiotensin-converting enzyme; D0, before transplantation; M1, M6 and M12, 1, 6 and 12 months post-transplantation, respectively

All transplantations were performed at the Leiden University Medical Center in the Netherlands. Transplantation procedures have previously been described [10]. Immunosuppressive therapies consisted of induction therapy followed by maintenance therapy, as previously described and indicated in Tables 1 and 2.

### Clinical variables

All patients and controls underwent routine venous blood sampling in the morning before intake of immunosuppression. Creatinine, haemoglobin, HbA_1c_, glucose, proteinuria in 24 h urine and urea were measured. GFR was calculated with plasma creatinine concentration using the Modification of Diet in Renal Disease (MDRD) formula. Plasma was harvested by centrifugation of EDTA-anticoagulated blood for 10 min at 1,000 *g* and subsequently stored at −80°C for VEGF and −20°C for MBL determination.

### *MBL2* genotyping

DNA was isolated from peripheral EDTA-anticoagulated blood. SNPs at codons 52 (D allele), 54 (B allele) and 57 (C allele) of the *MBL2* gene and in the promoter region were determined by high resolution melting analysis as previously described [12]. The ‘wild-type’ genotype is named A/A and the presence of 1 or 2 variant alleles(s) containing SNPs (B, C or D) are named A/O or O/O, respectively. The promoter region SNP, which results in reduced *MBL2* gene expression, has been named P.

### Plasma MBL and VEGF levels

Plasma MBL levels were assessed by ELISA as previously described [13]. VEGF levels were determined by ELISA (Diaclone Research, Besançon, France) according to the instructions of the manufacturer.

### Statistical analyses

In Table 1, parametric data are presented as mean ± SD. Nonparametric data are shown as median and interquartile range (IQR) and tested with one-way ANOVA (Tukey post hoc test). Categorical variables were analysed with Fishers exact test. The longitudinal study was analysed with repeated measures analysis. Differences between categorical data have been computed using Friedman’s 2-way ANOVA by ranks. Differences between MBL levels were adjusted for sex, age and duration of diabetes. Correlations were calculated using Pearson correlation. A *p* value <0.05 was considered to be statistically significant. Data analysis was performed using SPSS version 20.0 (SPSS, Chicago, IL, USA) and GraphPad Prism, version 5.0 (GraphPad Prism Software, San Diego, CA, USA).

## Results

### Circulating levels of MBL associate with diabetic nephropathy

**Fig. 1:**
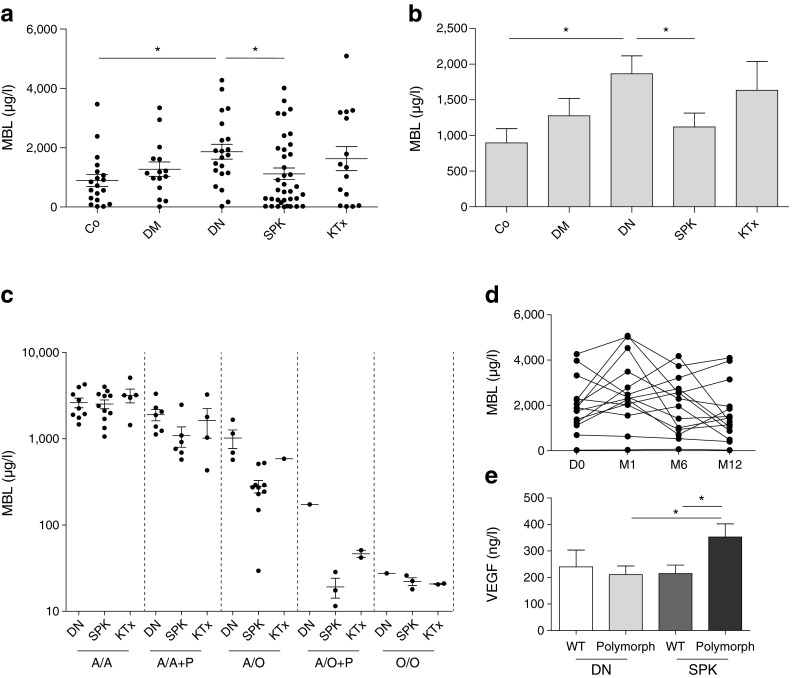
SPK in patients with type 1 diabetes reverses elevated MBL levels in association with *MBL2* genotype and VEGF expression. (**a**) Circulating MBL levels are increased in diabetic nephropathy (DN) patients and normalise after SPK. (**b**) Data in (**a**) presented as a bar graph. (**c**) Subdivision of polymorphisms per patient group suggests that predominantly patients with a polymorphism in the *MBL2* gene show normalisation of MBL levels after SPK. The scale is presented as a log_10_ scale. For an explanation of the different genotypes please refer to the ‘*MBL2* genotyping’ section of the Methods. (**d**) Circulating MBL levels in DN patients who received SPK and were followed up longitudinally before transplantation (D0), and 1, 6 and 12 months (M) after SPK. (**e**) VEGF levels plotted for DN and SPK groups divided by patients with wild-type MBL or MBL polymorphism carriers. *p < 0.05. Data are presented as mean + SEM. Co, control groups of healthy volunteers; DM, type 1 diabetes patients with an eGFR of ≥30 ml min^−1^ 1.73 m^−2^; DN, type 1 diabetic patients with diabetic nephropathy; KTx, diabetic nephropathy patients with a functioning kidney graft; SPK, patients who received SPK in the past

We first sought to determine whether diabetic nephropathy patients displayed differential levels of circulating MBL. As shown in Fig. 1a, diabetic nephropathy patients have significantly higher plasma MBL levels than healthy controls (*p* = 0.02) and a trend towards higher levels than type 1 diabetic patients with good renal function.

### Diabetic-nephropathy-associated circulating MBL levels normalise after SPK, but not after kidney transplantation alone

Given that SPK makes the patients normoglycaemic and restores kidney function, we sought to determine whether this would affect plasma MBL levels. As depicted in Fig. 1a, we observed that SPK normalised plasma MBL levels. In addition, we determined whether kidney transplantation alone would affect MBL levels. Interestingly, this did not alter MBL levels (Fig. 1a), suggesting that normoglycaemia is responsible for normalisation of MBL levels. Figure 1b illustrates average MBL levels among all patient groups. MBL significantly correlates with both levels of glucose (*p* = 0.005; *r* = 0.31) and HbA_1c_ (*p* = 0.02; *r* = 0.25), and also with duration of diabetes (*p* = 0.01; *r* = −0.26) and age (*p* = 0.006; *r* = −0.29). By contrast, we did not find significant correlations with creatinine levels and eGFR, or with proteinuria, retinopathy, neuropathy or cardiovascular events (data not shown).

### *MBL2* genotype affects normalisation of MBL levels after SPK

To investigate whether a specific *MBL2* genotype associated with SPK-mediated normalisation of circulating MBL levels, polymorphisms of the *MBL2* exon 1 and promoter were determined. We separated the patient groups per polymorphism and displayed the corresponding MBL levels (Fig. 1c), which illustrated that normalisation of MBL levels after SPK was particularly observed in patients with an MBL polymorphism, whereas it was less obvious in ‘wild-type’ MBL carriers.

### Longitudinal study shows a trend towards normalisation of MBL levels after SPK

Given that MBL levels show considerable variation among individuals, which complicates the interpretation of MBL differences, we next sought to determine if we could validate the normalisation of MBL levels in patients who were followed up after SPK. Although SPK seems to cause an initial increase in MBL levels at M1, 12 months after SPK we found a trend towards decreased plasma MBL levels (Fig. 1d).

### MBL levels associate with VEGF levels in a polymorphism-dependent manner

When patients were divided into wild-type MBL and MBL polymorphism groups, it was clear that VEGF levels increased after SPK in patients with an MBL polymorphism (Fig. 1e). This separation of *MBL2* genotypes also revealed a correlation of HbA_1c_ with VEGF levels (data not shown): in wild-type MBL carriers we observed a positive correlation, while in MBL polymorphism carriers there was a negative correlation. No *MBL2*-genotype-dependent correlations were observed with proteinuria, retinopathy, neuropathy or cardiovascular events.

## Discussion

In this study, we have shown an elevation of plasma MBL levels in patients with diabetic nephropathy, which normalises after SPK. Kidney transplantation alone does not result in a decrease in MBL levels, suggesting that this normalisation is dependent on glycaemic control.

Our data suggest that predominantly patients with an MBL polymorphism have normalised levels of MBL after SPK. Interestingly, low pretransplantation MBL levels have been shown to predict superior patient and graft survival after SPK [13]. Although speculative, our data suggest a link between high MBL levels and the lack of capacity to lower MBL levels after transplantation. We also found a relationship between MBL levels and circulating VEGF levels. Surprisingly, this correlation was positive in wild-type MBL carriers and negative in patients with an MBL polymorphism. A similar correlation was observed for VEGF with HbA_1c_ levels. Although (podocyte-derived) VEGF is usually considered to mediate the development of diabetic nephropathy [14], it has also been described that VEGF can be protective in diabetic nephropathy [15], providing a possible explanation for ‘better’ normalisation after SPK in patients with an MBL polymorphism. However, as group sizes were limited in our study, these observations need confirmation in larger patient cohorts.

In the longitudinal study, we observed a trend towards decreased MBL levels 12 months after SPK that would confirm the effect of SPK on MBL in patients in a controlled cohort with similar immunosuppressive drugs. This decrease was not as strong as we observed in the cross-sectional cohort. However, 12 out of 14 patients in the longitudinal study were wild-type MBL carriers (data not shown), which we, in the cross-sectional study, found to associate with only a modest decrease in MBL levels after SPK.

We cannot exclude that the use of immunosuppression after transplantation influenced the normalisation of MBL levels after SPK in our study. Nonetheless, this seems unlikely because patients in the KTx group also received immunosuppressive drugs but did not show decreased levels of MBL.

Taken together, we have demonstrated that circulating levels of MBL are elevated in diabetic nephropathy patients and normalised after SPK. The normalisation of MBL levels was independent of renal function, but dependent on glycaemic control, and might only occur in patients with a polymorphism in MBL, which could affect the development of vascular injury.

## Abbreviations

eGFR: Estimated GFR
IQR: Interquartile range
KTx: Kidney transplantation
MBL: Mannan-binding lectin
SNP: Single nucleotide polymorphism
SPK: Simultaneous pancreas–kidney transplantation
VEGF: Vascular endothelial growth factor
